# Five-Animal Frolics Exercise Improves Anxiety and Depression Outcomes in Patients with Coronary Heart Disease: A Single-Blind Randomized Controlled Trial

**DOI:** 10.1155/2020/6937158

**Published:** 2020-08-07

**Authors:** Jun Jiang, Qingbao Chi, Yuting Wang, Xue Jin, Shui Yu

**Affiliations:** ^1^Department of Cardiovascular, The First Hospital of Jilin University, Changchun 130021, China; ^2^Department of Spine Surgery, The First Hospital of Jilin University, Changchun 130021, China

## Abstract

**Introduction:**

The patients with coronary heart disease (CHD) always have emotional implications. As the branch of traditional Chinese medicine, Five-Animal Frolics Exercise (FAE) is a popular mind-body exercise in China and shown to improve emotional wellbeing.

**Aim:**

We aimed to explore the effects of FAE on the emotional disorders of CHD patients.

**Methods:**

CHD patients were assigned into an experiment group (EG, FAE) and a control group (CG, routine nursing care). We measured serum levels of miR-124 and miR-135 and scores of the Hamilton Depression/Anxiety scale (HAMD/HAMA), Self-Rating Anxiety Scale (SAS), the Self-Rating Depression Scale (SDS), Short Form 36 Health Survey Questionnaire (SF-36), and Pittsburgh Sleep Quality Index (PSQI).

**Results:**

After a 3-month FAE intervention, serum levels of miR-124 and miR-135 and the scores of HAMD/HAMA, SAS, SDS, and PSQI in the EG group were lower than those in the CG group, while SF-36 scores in the EG group were higher than those in the CG group (*p* < 0.05). Serum levels of miR-124 and miR-135 had a strong relationship with SAS and SDS scores (*p* < 0.05). *Discussion/Implications for Practice*. The study suggests that FAE intervention controls anxiety and depression outcomes and improves life quality in CHD patients by affecting serum levels of miR-124 and miR-135.

## 1. Introduction

As a traditional medical model shifts to a social-psychological-biological model of modern medicine, much attention is paid to the effects of psychological stress and mental disorders on coronary heart disease (CHD) [1, 2]. Psychological stress affects the neuroendocrine system and immune system, which in turn acts on the nervous system, causing anxiety and depression-like behaviors [3]. Psychological intervention has become the focus in the prevention of emotional disorders in heart disease patients [4]. Aerobic exercise can reduce the risk of adverse cardiovascular events in CHD patients and reduce the severity of anxiety and depression [5]. Exercise therapy can effectively improve anxiety, depression, and life quality. Furthermore, exercise therapy can reduce the levels of various inflammatory and cardiovascular indicators, and some scholars believe that exercise therapy also has a certain beneficial effect on neuroimmune [6].

As a branch of traditional Chinese medicine, Five-Animal Frolics Exercise (FAE) follows the social-psychological-biological model of modern medicine and coordinates the limbs and body by imitating animal gait [7]. It mainly prevents the progression of cardiovascular diseases by paying much attention to patients' mental and psychological problems and subjective feelings, emphasizing comprehensive treatment, and treating patients from multilevel and multiangle cooperation. The mind-body exercise is performed in a comprehensive and harmonious way. FAE not only treats patients with exercise treatment [8] but also pays attention to the changes of patients' psychological state and the negative effects caused by psychosocial situation [9]. miRNAs play a crucial role in the regulation of psychological stress reaction [10]. Exercise has been found to show a positive impact on psychological stress by decreasing miR-124 [11]. MiR-124 has been observed to play important roles in the etiology of depression [12]. MiR-135 is involved with NMDA receptor-dependent long-term depression [13] and a kind of anxiety-regulating microRNAs [14]. In this study, the effects of FAE on depression and anxiety of CHD patients were explored, and the changes of serum levels of miR-124 and miR-135 were investigated.

## 2. Patients and Methods

### 2.1. Design

This study belongs to a single-blind randomized trial. All procedures were approved by human research ethics committee of our hospital and conform to the provisions of the Declaration of Helsinki [15]. Informed consent to participate in the study was obtained from all participants.

### 2.2. Subjects and Recruitment

The cases included in this study were the patients who were discharged at our hospital from March 1, 2017, to March 1, 2018.

#### 2.2.1. Inclusion Criteria

The patients who met diagnostic criteria for CHD, anxiety, and depression were included. CHD was diagnosed according to the “Diagnostic Criteria for Coronary Atherosclerotic Heart Disease” issued by the Ministry of Health of the People's Republic of China in 2010; the diagnostic criteria for anxiety and depression refers to the Chinese Classification of Mental Disorders (Revision 3, CCMD-3) [16]. Anxiety and depression were measured based on the Hamilton Depression Rating Scale (HAMD) and the Hamilton Anxiety Scale (HAMA). They met the diagnostic criteria of anxiety and depression according to the HAMD, HAMA, Self-Rating Anxiety Scale (SAS), and Self-Rating Depression Scale (SDS).

#### 2.2.2. Exclusion Criteria

The patients who could not take some exercise and did not provide consent written form or history clinical data were excluded.

### 2.3. Sample Size Calculation

The sample size estimation of this study was due to the fact that there are few clinical studies on the therapy based on a FAE. Therefore, considering the sample size of each group, 100 cases of the lowest sample size of clinical research were the initial targets. Based on the actual situation, two aspects were considered. According to the estimated number of inpatients in our hospital, it was estimated that the number of patients who were diagnosed with CHD and met the inclusion and exclusion criteria in the annual inpatients was more than 100. The subjects who voluntarily joined the clinical study may be lower than the 100 cases. According to the period of the clinical study, the total duration was one year, and the follow-up period was 3 months; the shedding rate was set at 20%. Therefore, the final sample size of each group was 60, and the final number of total sample size was 120.

### 2.4. Randomization

**Figure 1:**
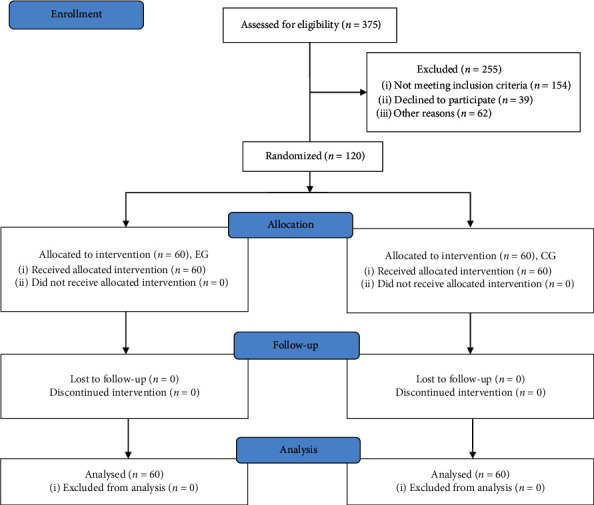
CONSORT flow diagram. EG, the patients received FAE. CG, the patients received routine nursing care. The follow-up was three months.

A total of 120 patients were assigned into the control group (CG, normal nursing care) and the experimental group (EG, FAE intervention), and the allocation ratio was 1 : 1 according to a random number produced by computer (Figure 1). Random allocation sequence was not concealed until interventions were assigned. Two investigators enrolled participants, and two statistical experts assigned participants to interventions. The exercise trainers were blinded after assignment to interventions. After admission, CHD patients were given conventional treatment in the CG group, such as the use of nitrates, anticoagulants, receptor blockers, vasoconvertase inhibitors, calcium antagonists, statins, and other drugs. Patient's psychological state should be understood, and negative mood was eliminated to avoid all kinds of incentives. The following items were observed: heart rate and pain location. After the pain of CDH patients was stable, appropriate physical exercise could be performed safely.

The therapy was performed in the EG group based on conventional treatment of the CG group. FAE were trained as follows: training time was selected at 4 pm daily during the patient's hospital stay. The location was selected in the open space of ward corridor. The exercise video was provided by our hospital for Loop Playback. The patients in the EG group were guided by five presentational trainers to learn FAE and mimic the movements of five different animals (tiger, deer, bear, monkey, and bird) and focused on massaging and strengthening specific internal organs. The patient's breath required a nasal suction, when the inhalation touched the upper tip of the tongue, the tongue tip was flat when exhaling, and the respiratory rate was 8–10 beats/min. The patients were practiced regularly, and meditation time was 30–60 min at one time with low-decibel meditation light music background and professional guidance.

### 2.5. Test Cycle and Visit Point

The test periods were 3 months in total, including 1 month of treatment and 3 months of observation. Follow-up was performed at 1 month and 3 months after the enrollment. Weekly telephone follow-up was performed to strengthen the subject's compliance and minimize the subject. The rate of dropout of the tester was also promptly excluded from subjects with poor compliance. There were 4 on-site visits at the month 0, 1, and 3. The telephone interviews determined the number of monthly telephone follow-ups based on the subject's compliance at our hospital.

### 2.6. Observation Index

General conditions included the current medical therapy, history, diagnosis, vital signs, physical examination, and the type, amount, and start and end time of the combined medication. Safety indicators included blood routine, urine routine, liver and kidney functions, blood lipids, blood sugar, BNP, 6-minute walk test, and Brog sensor score. Disease evaluation indicators included the HAMD, HAMA, Self-Rating Anxiety Scale (SAS), the Self-Rating Depression Scale (SDS), Short Form 36 Health Survey Questionnaire (SF-36), and Pittsburgh Sleep Quality Index (PSQI).

The statistical scale was measured at baseline and after 1 month and 3 months of treatment, and the amount of change in the score and the improvement rate were calculated; the improvement rate was given as (pretreatment score−treatment score)/pretreatment score × 100%; total effective rate = cure rate + significant efficiency + effective. The following criteria were used: recovery, improvement rate of 75%; markedly effective, 50% < improvement rate < 75%; effective, 25% < improvement rate < 50%; and invalid, improvement rate <25%.

### 2.7. Adverse Events

If an adverse event occurred, it should be recorded in time, and the corresponding method was taken according to the severity of the adverse event. The final result was recorded and analyzed if the adverse event disappeared. They should be reported to the responsible person for discussion and treatment if serious adverse events occurred.

### 2.8. Quantitative Real-Time PCR (qRT-PCR) Analysis

5 mL blood was obtained from each participant, and serum was prepared via centrifugation at 2000 × *g* for 10 min. Total RNA was obtained by using the RNA purification kit (Epicenter, Chicago, IL, USA). 2 *μ*g of RNA from each person was reversely transcribed by using the cDNA Reverse Transcription Kit (Applied Biosystems, Foster City, CA, USA). The following primers were used for real-time PCR and synthesized by TaKaRa (Dalian, China), miR-124 (forward primer: 5′-CTAGTCTAGAGTCGCTGTTATCTCATTG. TCTG-3′ and reverse primer: 5′-CGCGGATCCTCT GCTTCTGTCACAGAATC-3′), miR-135 (forward primer: 5′-AGCATAATACAGCAGGCACAGAC-3′ and reverse primer: 5′-AAAGGTTGTTCTCCACTCTCTCAC-3′), and U6 snRNA (5′-CTCGCTTCGGCAGCACA-3′) as an internal control. The qRT-PCR reaction was performed using the following condition: 1 cycle of 95°C for 40 s, followed by 45 cycles of 95°C for 10 s, 60°C for 20 s, and 1 cycle of 60°C for 50 s, and maintained at 4°C. Relative gene levels were normalized to the level of U6 snRNA and calculated by using the 2^−ΔΔCT^ method.

### 2.9. Statistical Analysis

The data were analyzed by using SPSS 20.0 statistical software. Count data were examined by using the Pearson 2 test. The two groups were compared by the LSD method if the data were in accordance with the normal distribution and expressed by the mean standard deviation (S.D.); they were represented by median (P25 and P75) and analyzed by a nonparametric test if they were not normally distributed. The data were compared in a single group before and after treatment, and a paired sample *t*-test was used if the normal distribution was consistent. The Wilcoxon signed rank sum test was used if it was not normally distributed. The Pearson correlation coefficient test was used to explore the relationship between the relative levels of miR-124 or miR-135 and/or SAS and SDS scores. The primary outcomes included anxiety and depression scores at 1 month. Secondary outcomes were measured after a 3-month follow-up. The statistically significant test level was set at *p* < 0.05, and the confidence interval of the parameter was 95%.

## 3. Results

### 3.1. Baseline Characteristics

In this study, no subjects were excluded from the study, and 120 subjects finished the present experiment, and the follow-up was 3 months (Figure 1). There was no significant difference in the baseline level between the two groups, including gender, age, combined disease (including hypertension, diabetes, and hyperlipidemia), blood lipids test (total cholesterol, glycerol triglyceride, and low-density lipoprotein), fasting blood glucose, electrocardiogram, BNP, the 6-minute walk test, and the Brog sensory score (Table 1, *p* > 0.05).

### 3.2. Anxiety and Depression Scale

HAMD, HAMA, SAS, and SDS were measured in the two groups. The anxiety and depression scales were scored at baseline (month 0), the first follow-up (month 1, primary outcomes), and the last follow-up (month 3, secondary outcomes). The first follow-up and baseline were calculated as the amount of change (I). The change in the final follow-up from the baseline was calculated as the amount of change (II).

Before intervention, the statistical difference for HAMD, HAMA, SAS, and SDS was insignificant between the two groups (*p* > 0.05, Table 2). After FAE intervention, the scores for HAMD, HAMA, SAS, and SDS in the EG group were lower than those in the CG group (*p* < 0.05, Table 2). The comparison of the amounts of changes (I) and (II) showed that the changes for the scores of HAMD, HAMA, SDS, and SAS in the EG group were higher than that in the CG group at baseline (month 0), the first follow-up (month 1), and the last follow-up (month 3) (Table 2, *p* < 0.05).

### 3.3. PSQI

The PSQI scores of the two groups were recorded at baseline (month 0), the first follow-up (month 1), and the last follow-up (month 3). The statistical difference for PSQI scores at baseline was insignificant between the two groups (Table 3, *p* > 0.05). After FAE intervention, the changes in PSQI scores were significantly different when compared with before intervention (Table 3, *p* < 0.05). The sleep quality of both groups was significantly improved. Baseline changes were compared between the groups and showed that the statistical differences were significant for the PSQI in amounts of changes (I) and (II) (Table 3, *p* < 0.05).

### 3.4. Comparison of Curative Effects

The percentage of total effectiveness of the PSQI efficacy of the two groups is described in Table 4. After FAE intervention, the EG group had a total effective rate of 100%, and the CG group had a total effective rate of 45% (*p* < 0.05). Comparing the total efficiency and inefficiency of the two groups, the statistical difference for the efficacy of PSQI was significant between the two groups.

### 3.5. Quality of Life Scale (SF-36)

The scores of the simple quality of life scale (SF-36) of the two groups were measured at baseline (month 0), the first follow-up (month 1), and the last follow-up (month 3). Except for body pain, the scores of the other factors were insignificant before intervention (Table 5, *p* > 0.05). The baselines of change (I) were statistically different for physical role functioning, vitality, emotional wellbeing, and mental health between the two groups (Table 5, *p* < 0.05) but not for other factors (Table 5, *p* > 0.05). The baselines of change (II) were statistically different for physical functioning, physical role functioning, emotional wellbeing, and perception of changes in health between the two groups (Table 5, *p* < 0.05) but not for other factors (Table 5, *p* > 0.05).

### 3.6. Laboratory Inspection and Other Indicators

All subjects had no abnormalities in both groups during blood tests, urine routine, and liver and kidney function tests during the clinical study. Combined with BNP, the 6-minute walk test, and Brog sensory score at baseline (month 0), the subjects of two groups had better cardiac function and performed the exercises or regular exercise (walking, cycling, and jogging.).

### 3.7. Number of Adverse Events and Treatment

During the 0-week period, the number of adverse events was two, the symptom was diarrhea, one was from the EG group, and the other occurred during the taking medicine. The tester did not have any further adverse events. There were no serious adverse events between the two groups.

### 3.8. FAE Reduced Serum Levels of miR-124 and miR-135 in CHD Patients

**Figure 2:**
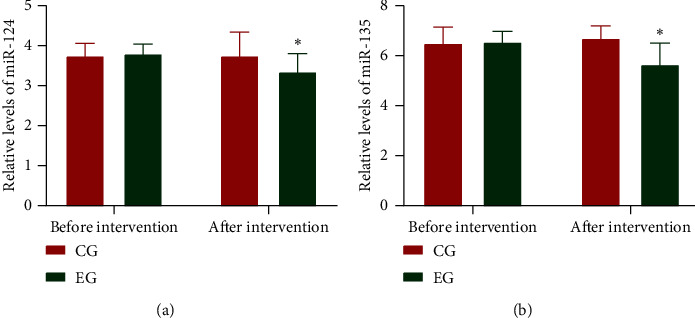
Relative levels of miR-124 and miR-135 in the patients with coronary heart disease. EG and FAE were used. CG, a control group. *n* = 20 for each group. The statistical different was significant if *p* < 0.05.

Before FAE intervention, the statistical difference for miR-124 (Figure 2(a)) and miR-135 (Figure 2(b)) was insignificant between the two groups (*p* > 0.05). After a 3-month intervention, the levels for miR-124 (Figure 2(a)) and miR-135 (Figure 2(b)) in the TG group were lower than those in the CG group (*p* < 0.05). The results suggest that FAE reduced serum levels of miR-124 and miR-135 in CHD patients.

### 3.9. Serum Levels of miR-124 and miR-135 had a Strong Relationship with SAS and SDS

**Figure 3:**
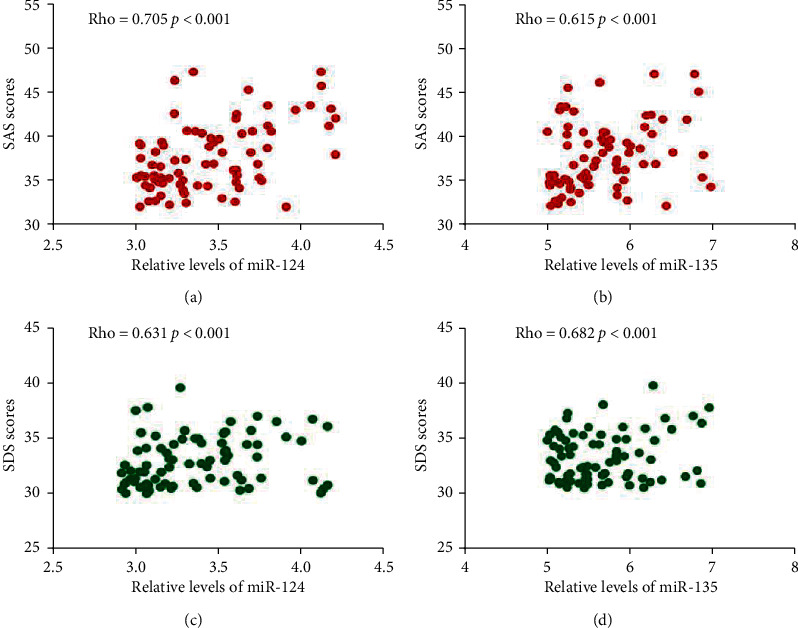
Pearson correlation coefficient analysis of the relationship between relative levels of miR-124 and miR-135 and the values of SAS and SDS. (a) The relationship between relative levels of miR-124 and the scores of SAS. (b) The relationship between relative levels of miR-135 and the scores of SAS. (c) The relationship between relative levels of miR-124 and the scores of SDS. (d) The relationship between relative levels of miR-135 and the scores of SDS. There is a strong positive relationship if rho value falls within 0.5 and 1. There is a strong negative relationship if rho value falls within −0.5 and −1.

The Pearson correlation coefficient test showed that the increase in the levels of miR-124 and miR-135 would result in the increase in the scores of SAS (Figures 3(a) and 3(b)), SDS (Figures 3(c) and 3(d), *p* < 0.05). The results suggest that serum levels of miR-124 and miR-135 have a strong positive relationship with SAS and SDS scores (*p* < 0.05).

## 4. Discussion

The differences for the changes of HAMD, HAMA, SDS, SAS, and PSQI scores were significant between the two groups (*p* < 0.05). The improvement in the EG group was better than that in the CG group. The results demonstrated that FAE had high efficacy for controlling the symptoms related to depression and anxiety in CHD patients. It may be a potential approach in the prevention of the progression of anxiety and depression in CHD patients.

After a 3-month intervention, serum levels of miR-124 and miR-135 and the scores of the HAMD/HAMA, SAS, SDS, and PSQI in the EG group were lower than those in the CG group, while SF-36 scores in the EG group were higher than those in the CG group. Serum levels of miR-124 and miR-135 had a strong positive relationship with SAS and SDS. The results suggest that miR-124 and miR-135 may be candidate biomarkers in the diagnosis of the CHD patients with depression and anxiety. The study suggests that FAE can control the symptoms related to anxiety and depression and improve sleep quality and life quality in CHD patients by affecting serum levels of miR-124 and miR-135. The results were consistent with a previous report that FAE intervention improved anxiety and emotional disorders in the patients [17].

According to the anxiety and depression scores, the patients had different degrees of mild-to-moderate anxiety and depression before the intervention. After the implementation of FAE, the anxiety and depression of patients were alleviated and improved, further demonstrating that psychological intervention promoted the mental health of patients. The number of anxiety and depression in the two groups decreased after intervention, but the number of anxiety and depression in the EG group was significantly lower than that in the CG group (*p* < 0.05), indicating that the implementation of the FAE reduces more anxiety and depression than normal physical exercise.

There is increasing strong evidence supporting the integrated effectiveness of mind-body treatments (nonpharmacological interventions) for heart disease [18]. Mindfulness-based meditation can attenuate the severity of depression and anxiety symptoms among heart disease patients and improve depression and anxiety clinical outcomes. FAE is a Chinese traditional Qigong, which focuses on mind-body integration, and thought to be effective in improving physical and mental wellbeing. FAE has been found to be effective in the prevention of the progression of anxiety and depression in chronic obstructive pulmonary disease (COPD) patients [19]. FAE is figuratively known as the “five animals” exercise, which is the most interesting activity for most CHD patients. CHD patients will be glad to participate in the activities, which show the moderating effect of mindfulness and benefits on multidimensional mental health [20]. FAE maintains single-minded status and effectively eliminates the distractions, which are harmful to mental health [21]. All these properties of FAE may contribute to alleviate the pathogenesis of emotion disorders of CHD patients.

### 4.1. Study Limitations

There were some limitations in the present work. The subjects were only included at our hospital, which had certain geographical restrictions, which led to some selection biases. The four anxiety scales used in this study were credible in the psychiatric reliability. However, for nonpsychiatric diseases and nonpsychiatric physicians, their reliability may be affected because they were not analyzed by psychiatrists, and the diagnosis of emotional disorders only included anxiety and depression. FAE is only popular in China and seldom known in other countries. The detailed molecular mechanism on how FAE can affect miRNA remains unclear. In the comparison of life quality of SF-36 between two groups, the difference of the amounts of change (I) of vitality and mental health was statistically significant between the EG group and the CG group. But the difference of the amounts of change (II) of vitality and mental health between the EG group and the CG group was statistically insignificant. The reasons may be caused by the following reasons: (1) the study was performed in the small population, and some bias will be induced (*n* = 60 in each group); (2) one and three months of intervention still belongs to a short-term study. A long-term study, such as one-year term, may be necessary to find the stable results for mental health [22]. A short-term intervention may lead to a significant variety of mental health issue, just as the outcome of one month of intervention may be occasionally better than that of three months of intervention in the present result. Vitality is closely associated with mental health and can be defined by using mental wellbeing [23], and a short-term study will cause the results in a significant variety. Therefore, a long-term study in a larger population is possibly also needed to find the stable results.

## 5. Conclusions

The EG group has better improvement in the anxiety and depression scale (HAMD, HAMA, SAS, and JDS), PSQI, curative effects, and SF-36. Serum levels of miR-24 and miR-135 had a strong positive relationship with SAS and SDS scores. The study suggests that FAE inhibits the risk of anxiety and depression and improves sleep quality and life quality in CHD patients by affecting serum levels of miR-24 and miR-135. We recommend that the CHD patients should insist on practicing FAE to prevent the risk of mental diseases, such as depression and anxiety. On the other hand, a long-term study in a larger population is needed to confirm the present conclusion.

## Figures and Tables

**Table 1:** Clinical baseline characteristics between two groups.

Parameters	EG (*n* = 60)	CG (*n* = 60)	*p* values
Gender (male)	24 (40%)	24 (40%)	—
Age (yr)	61.00 ± 8.93	63.10 ± 10.04	0.763
Combined with hypertension	45 (75%)	42 (70%)	0.816
Combined with diabetes	24 (40%)	24 (40%)	—
Combined with tuberculosis	57 (95%)	51 (85%)	0.615
Abnormal ECG check	33 (55%)	36 (60%)	0.805
Total cholesterol	3.89 ± 1.32	3.82 ± 1.03	0.965
Triglyceride	1.50 ± 0.92	1.82 ± 0.78	0.328
Low-density lipoprotein	2.04 ± 1.06	1.91 ± 0.63	0.877
Fasting blood sugar	6.60 ± 1.40	7.28 ± 2.62	0.372
BNP	87.02 ± 79.40	99.96 ± 138.24	0.868
6-minute walk test	527.78 ± 49, 77	539.25 ± 57.59	0.135
Brog feels the score	11.89 ± 1.61	11.25 ± 1.68	0.409

**Table 2:** Comparison of four anxiety depression scales between two groups.

Parameters and time points		EG	CG	*p*
HAMD	0 month	12.0 (10.0, 14.0)	11.5 (8.3, 14.8)	0.908
	1 month	7.5 (5.3, 10.0)	9.5 (7.0, 12.0)	
	3 months	5.0 (4.0, 6.0)	8.0 (5.3, 10.0)^#^	
	Amounts of change (I)	4.0 (2.3, 6.8)^#^	2.0 (0.3, 3.8)	0.015^*∗*^
	Amounts of change (II)	7.0 (5.3, 8.8)^#^	3.0 (2.3, 5.8)	0.002^*∗*^

HAMA	0 month	17.0 (15.0, 19.0)	15.0 (12.0, 17.0)	0.095
	1 month	10.0 (7.0, 12.0)	12.0 (10.0, 14.0)	
	3 months	5.5 (5.0, 7.8)^#^	9.0 (7.0, 12.0)^#^	
	Amounts of change (I)	8.0 (5.3, 9.0)^#^	2.0 (2.0, 4.0)	<0.001^*∗*^
	Amounts of change (II)	11.0 (9.0, 12.0)^#^	5.0 (4.0, 7.8)	<0.001^*∗*^

SDS	0 month	41.0 (39.3, 47.5)	40.5 (35.3, 49.0)	0.770
	1 month	34.0 (31.0, 36.0)	35.0 (32.0, 44.3)	
	3 months	30.0 (29.3, 32.0)^#^	34.0 (30.3, 40.8)^#^	
	Amounts of change (I)	7.0 (5.0, 10.0)^#^	3.0 (1.0, 6.8)	0.012^*∗*^
	Amounts of change (II)	10.0 (8.3, 14.8)^#^	5.5 (3.3, 8.0)	0.005^*∗*^

SAS	0 month	50.0 (51.0, 55.5)	50.0 (50.0, 52.0)	0.106
	1 month	41.0 (34.0, 43.5)	45.0 (39.5, 48.8)	
	3 months	32.0 (31.0, 37.5)^#^	40.0 (32.8, 44.3)^#^	
	Amounts of change (I)	14.5 (10.3, 18.0)^#^	4.5 (3.0, 8.8)	<0.001^*∗*^
	Amounts of change (II)	20.0 (15.3, 22.8)^#^	8.5 (6.3, 17.0)^#^	<0.001^*∗*^

*Note.* Amounts of change (I) is the comparison between 1 month and 0 month, and the amounts of change (II) is the comparison between 3 months and 0 month. ^*∗*^The comparison between the test group and the control group is statistically significant (*p* < 0.05). ^#^There were significant differences between the two groups before and after treatment (*p* < 0.05).

**Table 3:** Comparison of PSQI between two groups.

	EG	CG	*p* values
0 month	14.0 (10.3, 15.0)	11.0 (9.0, 13.8)	0.100
1 month	9.0 (6.5, 11.8)	9.0 (7.3, 12.0)	
3 months	7.0 (5.0, 7.8)^#^	8.0 (6.0.1 5)^#^	
Amounts of change (I)	3.0 (1.3, 6.8)^#^	1.0 (0.0, 1.8)	0.003^*∗*^
Amounts of change (II)	6.0 (4.0, 8.0)	2.5 (1.0, 4.0)^#^	0.001^*∗*^

*Note.* Amounts of change (I) is the comparison between 1 month and 0 month, and the amounts of change (II) is the comparison between 3 months and 0 month. ^*∗*^The comparison between the test group and the control group is statistically significant (*p* < 0.05). ^#^There were significant differences between the two groups before and after treatment (*p* < 0.05).

**Table 4:** Comparison of curative effect of PSQI.

Groups	Therapeutic results				Total effective	*p* values
	Healing	Significant effective	Effective	Invalid		
EG		7 (35%)	13 (65%)		100%^#^	<0.001^*∗*^
CG	1 (5%)	2 (10%)	6 (30%)	11 (55%)	45%	

*Note.* ^*∗*^The comparison between the test group and the control group is statistically significant (*p* < 0.05). ^#^There were significant differences between the two groups before and after treatment (*p* < 0.05).

**Table 5:** The comparison of life quality of SF-36 between two groups.

Parameters and time points		EG	CG	*p*
Physical functioning	0 month	88.0 (84.0, 100.0)	85.0 (85.0, 95.0)	0.430
	1 month	94.0 (88.0, 100.0)	85.0 (80.0, 95.0)	
	3 months	100.0 (100.0, 100.0)^#^	87.5 (81.3, 95.0)^#^	
	Amounts of change (I)	3.8 (0.0, 5.0)^#^	0.0 (0.0, 0.0)	0.295
	Amounts of change (II)	5.0 (0.0, 10.0)	0.2 (0.0, 5.0)	0.003^*∗*^

Physical role functioning	0 month	12.9 (0.0, 18.8)	11.3 (0.0, 75.0)	0.925
	1 month	25.0 (25.0, 50.0)	0.0 (0.0, 87.5)	
	3 months	50.0 (50.0, 100.0)^#^	37.5 (6.3, 100.0)^#^	
	Amounts of change (I)	21.0 (0.0, 25.0)^#^	0.0 (0.0, 0.0)	0.049^*∗*^
	Amounts of change (II)	50.0 (25.0, 50.0)^#^	12.5 (0.0, 43.8)	0.003^*∗*^

Body pain	0 month	84.0 (75.0, 100.0)	79.0 (74.0, 100.0)	0.042^*∗*^
	1 month	95.0 (88.0, 100.0)	84.0 (74.0, 100.0)	
	3 months	92.0 (74.0, 100.0)^#^	89.0 (76.5, 100.0)^#^	
	Amounts of change (I)	2.0 (0.0, 9.0)	0.0 (0.0, 0.0)	0.109
	Amounts of change (II)	3.1 (0.0, 15.0)	3.3 (0.0, 26.0)	0.274

General health perceptions	0 month	30.0 (25.0, 45.0)	32.5 (20.0, 40.0)	0.579
	1 month	45.0 (26.8, 53.8)	32.5 (25.0, 45.0)	
	3 months	56.0 (45.0, 70.3)^#^	52.5 (35.0, 67.0)^#^	
	Amounts of change (I)	3.6 (0.0, 13.8)	1.2 (0.0, 3.8)	0.637
	Amounts of change (II)	20.0 (5.5, 30.0)	17.0 (5.0, 30.3)	0.949

Vitality	0 month	45.0 (41.0, 50.0)	55.0 (45.0, 65.0)	0.117
	1 month	55.0 (50.0, 68.8)	55.0 (45.0, 65.0)	
	3 months	62.5 (55.0, 70.0)^#^	57.5 (51.3, 73.8)^#^	
	Amounts of change (I)	10.0 (0.0, 18.8)	0.0 (0.0, 0.0)	≤0.001^*∗*^
	Amounts of change (II)	15.0 (5.3, 23.8)	5.0 (0.0, 10.0)	0.073

Social functioning	0 month	88.0 (75.0, 100.0)	88.0 (65.3, 100.0)	0.610
	1 month	88.0 (88.0, 100.0)	88.0 (78.3, 100.0)	
	3 months	100.0 (100.0, 100.0)^#^	100.0 (100.0, 100.0)^#^	
	Amounts of change (I)	5.0 (0.0, 12.8)	0.0 (0.0, 0.0)	0.172
	Amounts of change (II)	12.0 (0.0, 13.0)	12.0 (0.0, 34.0)	0.728

Emotional wellbeing	0 month	0.0 (0.0, 91.8)	0.0 (0.0, 100.0)	0.942
	1 month	50.0 (33.0, 100.0)	0.0 (0.0, 100.0)	
	3 months	100.0 (67.0, 100.0)^#^	33.0 (8.3, 100.0)^#^	
	Amounts of change (I)	23.0 (0.0, 33.0)^#^	0.0 (0.0, 0.0)	0.012^*∗*^
	Amounts of change (II)	67.0 (8.3, 100.0)^#^	0.0 (0.0, 33.0)	0.003^*∗*^

Mental health	0 month	50.0 (42.0, 60.0)	48.0 (42.5, 56.0)	0.073
	1 month	60.0 (49.0, 68.0)	48.0 (42.5, 55.8)	
	3 months	68.0 (60.0, 72.0)^#^	56.0 (48.0, 64.0)^#^	
	Amounts of change (I)	8.0 (0.0, 18.5)^#^	0.0 (0.0, 0.0)	0.001^*∗*^
	Amounts of change (II)	17.0 (1.0, 24.0)	4 (−0.8, 13.5)	0.062

Perception of changes in health	0 month	25.0 (25.0, 25.0)	25.0 (25.0, 50.0)	0.666
	1 month	37.5 (25.0, 50.0)	25.0 (25.0, 50.0)	
	3 months	50.0 (25.0, 50.0)^#^	50.0 (25.0, 50.0)	
	Amounts of change (I)	0.0 (0.0, 25.0)	0.0 (0.0, 0.0)	0.322
	Amounts of change (II)	15.0 (0.0, 25.0)	0.0 (0.0, 25.0)	0.002^*∗*^

*Note.* Amounts of change (I) is the comparison between 1 month and 0 month, and the amounts of change (II) is the comparison between 3 months and 0 month. ^*∗*^The comparison between the test group and the control group is statistically significant (*p* < 0.05). ^#^There were significant differences between the two groups before and after treatment (*p* < 0.05).

## Data Availability

The data for the current study are available from the corresponding author upon reasonable request.
